# On the Hot-Plate Welding of Reactively Compatibilized Acrylic-Based Composites/Polyamide (PA)-12

**DOI:** 10.3390/ma16020691

**Published:** 2023-01-10

**Authors:** Henri Perrin, Masoud Bodaghi, Vincent Berthé, Sébastien Klein, Régis Vaudemont

**Affiliations:** Luxembourg Institute of Science and Technology (LIST), 5, rue Bommel, L-4940 Hautcharage, Luxembourg

**Keywords:** fusion bounding, Elium^®^, reactive welding

## Abstract

Joining of dissimilar thermoplastics and their composites is a challenge for thermal welding techniques due to different melting points. Reactive welding with an auxiliary functional material can offer the clear opportunities to develop joining processes due to robustness to joining dissimilar thermoplastic polymers and their composites. The current study employed reactive compatibilization to offer the possibility of joining an acrylic-based glass fiber composite to polyamide (PA)-12 by applying a hot-tool welding technique. For this purpose, composite plates are fabricated by a typical vacuum infusion and thin layer thermoplastic films are formed by a thermostamping of PA12 granules. Subsequently, the reactive welding of the interposed PA12 sheet and Elium^®^-GMA-Glass composite is conducted by hot-plate welding. A glycidyl methacrylate (GMA) as a compatibilizing agent is copolymerized with methyl methacrylate Elium^®^ resin. During the hot-tool welding process of dissimilar thermoplastic material, GMA can react with the polyamide end groups. The heat distribution at the Elium^®^ GMA/PA-12 interface is responsible for obtaining a strong joint. This study focuses on the functionality of the compatibilizer on the welding of acrylic-based composites with polyamide (PA)-12 while varying the assembly temperature. The flatwise tensile test proved the effectiveness of GMA on the interface bounding. The excellent bounding incompatible polymers Elium^®^ resin (PMMA) and PA12 was achieved at 200 °C.

## 1. Introduction

The versatile application of multi material design consisting of dissimilar polymers and their composites is a possible response to the increasing demand of reducing weight and improving mechanical properties in automotive and aerospace industries. In practical applications, joining light thermoplastic and high-strength fiber reinforced thermoplastic composite is a widespread design [1,2].

Fusion bounding is a highly practical technique for joining fiber reinforced thermoplastic composites. Experience with the fusion bounding method has proven the possibility of joining dissimilar parts with eliminating stress concentration and rivets while staying cost effective. In this family of processes, after close contact of surfaces, inter-diffusion over a period allows joining the two polymeric parts or their composites above the certain temperature. Depending on the heating mechanisms, several variants of fusion bounding, such as hot plates, ultrasonic, laser, induction, and friction stir, have been developed and matured [3].

In recent years, welding dissimilar thermoplastic materials has become a hot research topic and various techniques have been tried to acquire the robust joining. A few studies reported the fusion bounding for dissimilar thermoplastic materials with compatible molecular structures, including polyethylene (PE)-polypropylene (PP) [4], PA6-PA66 [5], polylactic acid-polyformaldehyde (PLA-POM) [6], PLA-poly (methyl methacrylate) (PMMA) [7], and PMMA-acrylonitrile butadiene styrene (ABS) [8]. These studies addressed proper welding strategies, such as welding time to ensure joint strength. So far, there are few reports on fusion bounding for incompatible polymers [9]. Fu et al. [9] successfully welded PP and PA6 by an interlayer solder sheet (ISS) prepared by blending of maleic anhydride-functionalized polypropylene (PP-g-MAH) and polyamide 6 (PA6) by an ultrasonic welding device. However, such thermoplastic systems must be processed at high processing temperatures, for example, PA-6 above 160 °C, PP in the temperature range of 230–270 °C [10]. Hence, their applications for manufacturing continuous fiber reinforced thermoplastic are limited in size and thickness due to their high melt viscosity [10].

One stablished way is a shift from melt processing to a reactive processing by using mono or oligomeric precursors, such as PA12 polymerized from *w*-laurolactam (Tm = 154 °C) using an initiator. The reactive processing of continuous fiber reinforced thermoplastic composite is performed at the temperature range of 90–250 °C. Hence, manufacturing of large composite parts, such as wind turbine blades, becomes very difficult [10]. Arkema developed an infusible thermoplastic based acrylic resin to manufacture composite parts at the temperature range of 25–90 °C depending on the variants of the acrylic resin [11]. This thermoplastic resin, known by its commercial name Elium^®^, possesses comparable in-plane mechanical properties to the high-performance epoxy resin after curing at room temperature [12]. Elium^®^ matrix fiber reinforced composites can be manufactured by using the Liquid Composite Molding family such as Resin transfer Molding and vacuum infusion processes thanks to their low viscosity range from 50 mPas to 100 mPas [11,12] at room temperatures. The components of Elium^®^ acrylic resin are 2-Propenoic acid, 2-methyl-, methyl ester or methylmethacrylate monomer (MMA), and acrylic copolymer. A radical initiator, such as a peroxide, is mixed with Elium^®^ acrylic resin at a particular weight ratio to convert MMA to its polymer PMMA under the in situ polymerization.

In recent years, the research on the fusion bounding of Elium^®^ composites, particularly ultrasonic welding, IR welding, and induction welding, has been conducted [13,14,15]. The temperature range at which it can be welded and the heating time are crucial parameters in the potential thermal degradation of the Elium^®^ composites during welding [16,17]. To enable the use of welding for the joining, this study for first time presents hybridization of Elium^®^ composites and a thin layer PA12 film by a reactive welding technique.

### The Significace of the Study

PMMA has a remarkable transparency, but due to a low glass transition temperature around (Tg) 110 °C and high sensitivity to solvents, its applications are limited. Particularly in fusion bounding process where the temperature of exposed surfaces reaches as high as 200 °C, there is the possibility of thermal degradation of PMMA. In general, amorph polymers or semi-crystalline ones should not be welded at a temperature above 75% of their glass transition point T_g_ or of their melting point T_m_, correspondingly [1]. There have been a few suggestions for the improvement of thermal stability of PMMA: 1- crosslinker agent: the weldability of Elium^®^ composites with crosslinker agents are reported elsewhere by the authors [18], 2: an interposed sheet of semi-crystalline or amorphous thermoplastic polymer. In the later process, the interposed polymer sheet acts like an adhesive. The interposed polymer sheet is stacked between two similar thermoplastic composites prior the consolidation process. During consolidation, the sandwich-like polymer layers are melted together and subsequently are bounded by intermolecular diffusion [1].

The incorporation of PA-12 in the form of an interposed polymer sheet between the PMMA fiber composite plates could make it possible to overcome the thermal degradation of PMMA at high temperatures while retaining the transparency of PMMA. Polyamide-12 (PA-12) not only has a high temperature resistance with the polymer melting point of 175 °C due to the presence of strong hydrogen bounds between strings, but also has a transmission, as well as a refraction index (around 1.5) equivalent to the PMMA. It should be noted that a desired through thickness interpenetration is highly influenced by the magnitude of the viscosity difference in the interposed polymer sheet and the thermoplastic matrix resin. In addition, for a successful bounding, the interposed polymer sheet should have compatible molecular structure with the polymer matrix [19]. The addition of a third component, namely a compatibilizing agent, is a common method to enhance the compatibility between dissimilar polymers.

Compatibilization is commonly used to obtain a stable polymer mixture. A compatibilizing agent (block copolymer or graft) is introduced or formed in situ at the interface from polymers bearing functions mutually reactive chemicals. The latter process is called reactive compatibilization. The compatibilizing agent located at the interface allows a good adhesion between the dissimilar polymers thanks to the entanglements of the components of the copolymer at the interface with the polymers. It also lowers interfacial tension and prevents coalescence. Glycidyl methacrylate monomer (GMA) has been successfully used as an efficient reactive compatibilizer for reactive polymer blending, such as poly (butylene terephthalate (PBT)/polypropylene (PP) [20], PP/polycarbonate (PC) [21], PP/Polyamide 6 [22]. The presence of an epoxy group in the GMA provides reactive sites, which enables copolymerizing with a variety of monomers for modifications such as crosslinking and interfacial adhesion. All compatibilizers, which are well-known for reactive blending, are not successfully implemented to the functionalization of a liquid resin thermoplastic due to the viscosity limitation for liquid composite molding, which must be below 0.5 Pa.s. In addition, the compatibilizers should not modify the fast-curing kinetics of the acrylic-based resin such as Elium^®^, which is well adopted for the liquid composite molding (LCM) process. Finding an effective compatibilizer for the functionalization of a liquid resin thermoplastic that can react at their welding interface is not a trivial task. However, no reactive welding has been reported for the above systems. To fully exploit the Elium^®^ thermoplastic composite design possibility, this study demonstrates the role of in situ functional Elium^®^-GMA copolymer composites.

**Figure 1:**
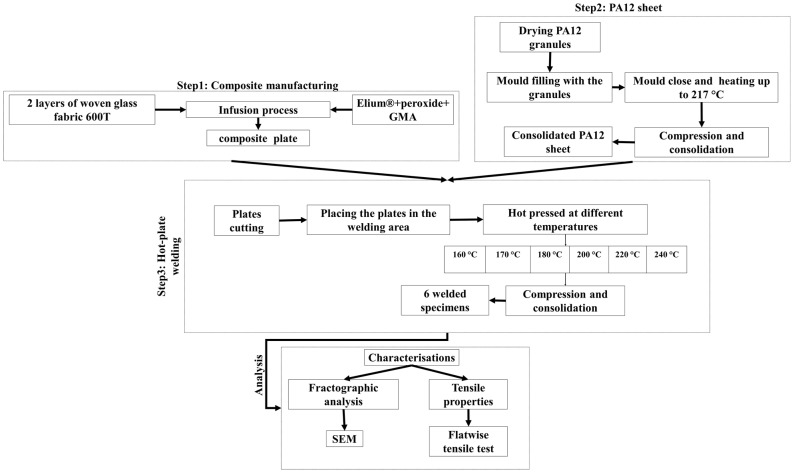
The experiment steps for hot-plate welding of reactively compatibilized acrylic-based composites/polyamide (PA)-12 material.

This study proposed the functionalization of acrylics-based resin by GMA for the liquid composite molding (LCM) of functionalized continuous fibers reinforced composites and the reactive welding. For this purpose, we manufacture composite plates by a typical vacuum infusion and PA12 sheets by a thermostamping technique. Subsequently, hot-plate welding is used for the reactive welding of the interposed PA12 sheet and Elium^®^-GMA-Glass composite. The variable involved in this study is temperature. The experiment steps are shown in Figure 1.

## 2. Materials and Methods

### 2.1. Fabrication Procedure

The welding was conducted at three separate steps. Step 1 was manufacturing glass fiber composite plates with an Elium^®^ matrix. Step 2 was manufacturing PA12 sheets. Step 3 was hot-tool welding.

#### 2.1.1. Composite Manufacturing

The Glass 600T, which is a continuous twill-weave glass fabric with an areal density of 600 g/m^2^ from CHOMARAT (Le Cheylard, France), was used. Two-layered preform was formed with 350 mm × 350 mm fabric plies, leading to a thickness around one mm after vacuum bagging. The atmospheric pressure pulled the mixture of the Elium^®^ 188XO resin with the initiator in the mass ratio of 100:2 and 5 wt% GMA (ref.779342 from Mercks, Branchburg, New Jersey, United States) from its reservoir into the preform by vacuum.

#### 2.1.2. Thermoplastic Polymer Sheets (PA12)

**Figure 2:**
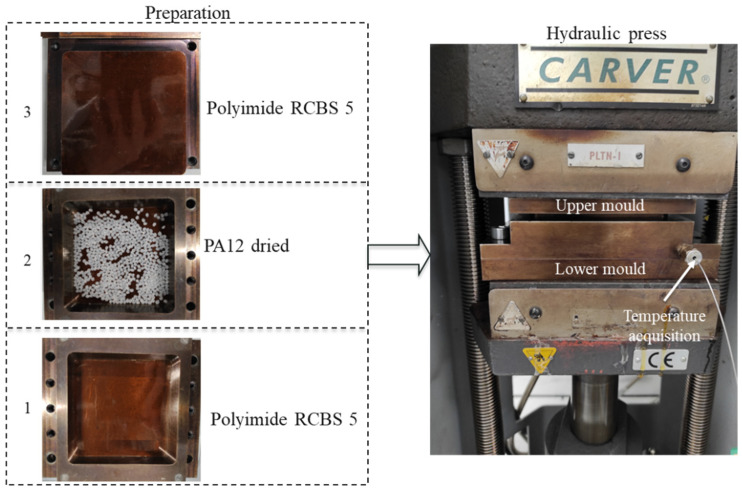
Preparation of PA12 sheet. 1- the lower mold covered by polyimide RCBS 5, 2- 12 g dried PA12, 3- upper mold covered by polyimide RCBS 5.

A compression molding machine (Figure 2 left) was used to melt and compress the pre-weighed PA12 granules from Arkema. The sheets were fabricated with the size 100 mm × 100 mm × 1.5 mm. The fabrication steps (Figure 2 right) started with drying PA12 granules at 80 °C for 12 h. Before filling the mold with 12 g dried PA12, the lower and the upper mold were covered by polyimide RCBS 5, which is a release film for thermoforming of thermoplastic materials up to 405 °C (see Figure 2). The mold was filled with PA12 granules and was subsequently closed. The process was followed with the mold heating to 217 °C to obtain the tool temperature of 200 °C. At 200 °C, a compression pressure of 0.5 MPa was applied and held for 10 min to achieve the desired sheet thickness. Finally, the consolidated PA12 sheet was demolded.

#### 2.1.3. Assembly of PA12 Sheets with Elium^®^ Composite Plates

**Figure 3:**
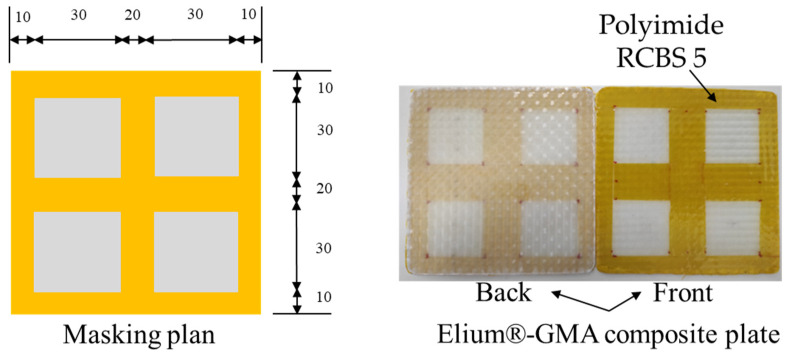
Masked Elium^®^-GMA glass fiber composite (EMG) film preparation. All dimensions are in mm.

For the assembly, the two 100-mm squared plates were cut from the manufactured Elium^®^ composite, and subsequently were masked with polyimide tape on the rough side (side faced with the vacuum bag) as shown in Figure 3. The masked plates were later dried at 80 °C for 12 h. Subsequently, the plates were cleaned with Diestone and dried again at 80 °C for 30 min.

**Figure 4:**
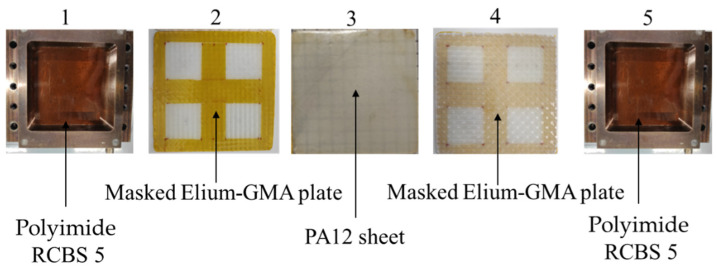
Sequence of layers arranged in the mold. 1- layer of polyimide RCBS5, 2- a masked Elium^®^-GMA plate, 3- a PA12 sheet, 4- a masked Elium^®^-GMA plate, 5- layer of polyimide RCBS5.

The assembly was started with stacking the first layer of polyimide RCBS5, a masked Elium^®^-GMA plate, a PA12 sheet, a masked Elium^®^-GMA plate, and the last layer of polyimide RCBS5 (Figure 4).

The entire assembly was hot pressed at a fixed compression pressure (0.5 MPa) and a fixed holding time (10 min). The temperature of hot-plate surfaces can be adjusted and was the only variable parameter to study the weldability of reactively compatibilized acrylic-based composites/polyamide (PA)-12 material (see Table 1).

### 2.2. Flatwise Tensile Test

**Figure 5:**
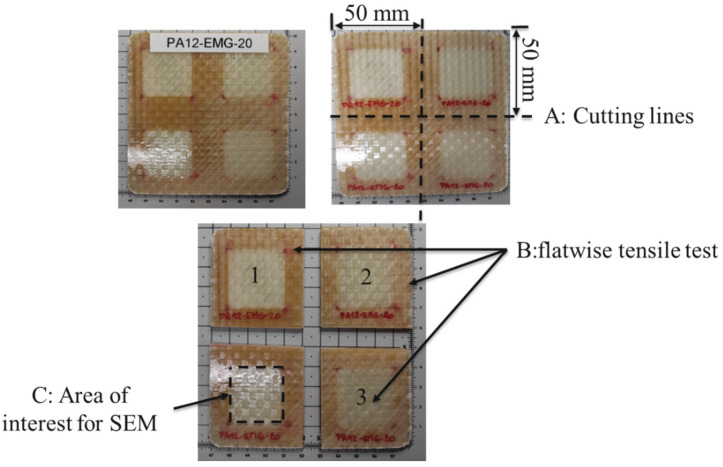
(**A**) Cutting configuration, (**B**) specimens for flatwise tensile test (1–3), (**C**) area of interest for Scanning Electron Microscopy (SEM).

Three specimens from each welded Elium^®^ composite laminate-GMA-PA12 were cut. The cutting configuration was shown in Figure 5A. The surface area of a specimen was 50 mm × 50 mm with an average thickness of 2.9 mm. For each of the welding scenarios (see Table 1), three iterations (Figure 5B 1–3) for the tensile test were carried out at repeatable conditions (at a temperature of 23 °C and a relative humidity of 50%).

**Figure 6:**
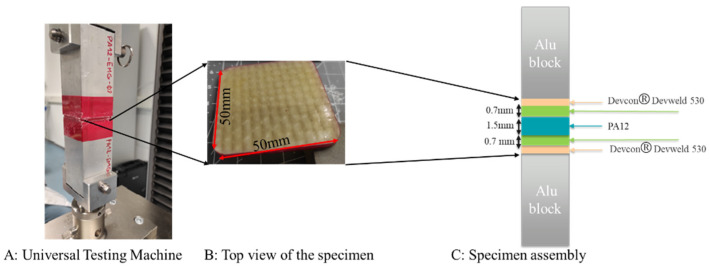
Schematic of assembled samples for flatwise test according to ASTM C297.

The flatwise tensile test was conducted on the specimen according to ASTM C297 [23]. The square specimen was loaded into the universal testing machine. Figure 6A shows the whole assembly setup for the experiment, while the assembled square sample configuration under stress through the thickness is given in Figure 6B,C. The load cell of the universal Instron machine for the tensile test was adjusted to 1 kN at the displacement rate of 2 mm/min. The data were recorded in terms of the maximum load and the load-displacement curve.

### 2.3. Fractographic Analysis

When the specimens are subjected to flatwise tensile test, fracture is cohesive, adhesive, or interfacial depending on the location of debonding [24]. To recognize the type of fracture, a fractographic analysis was carried out on the coupon surfaces using Scanning Electron Microscopy (SEM) (Figure 5C). SEM was a pressure-controlled FEI Quanta 200 FEG (Field Electron and Ion Company, FEI, Hillsboro, Oregon, United States). After mounting the samples in resin, they were polished with a diamond to achieve a sub-micron finish. Due to poor electrical conductivity, the samples were coated in a thin layer (about 10 nm) of gold.

## 3. Results and Discussion

### 3.1. Tensile Properties

**Figure 7:**
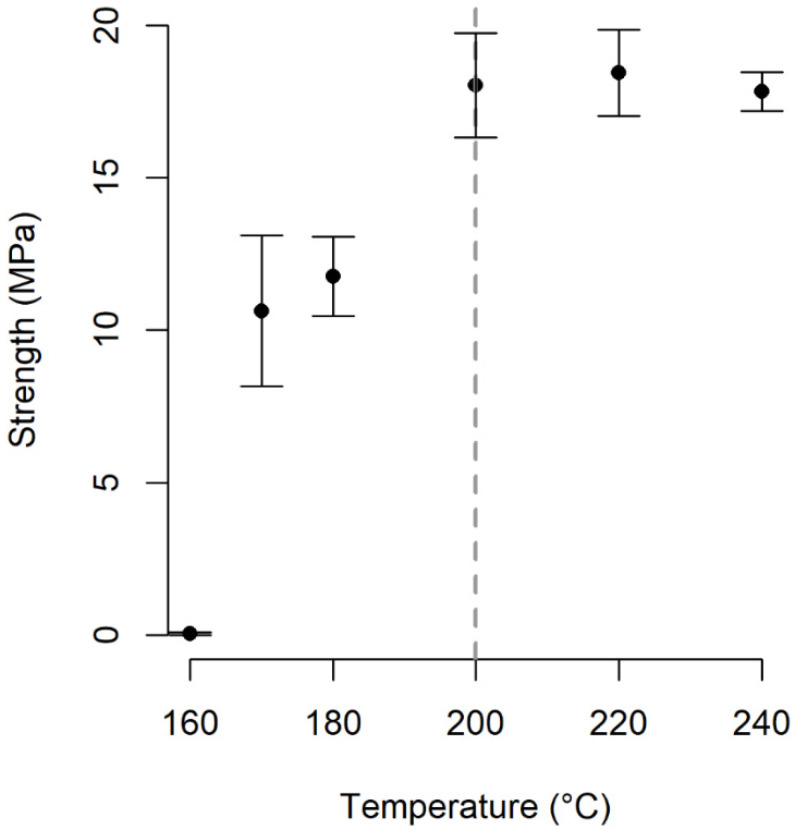
Variation of tensile strength versus heating temperature at constant holding time and pressure for PA12-EMG. EMG stands for Elium^®^-GMA-Glass composite.

As seen in Figure 7, at a constant hold time and constant pressure, the max load value increased with increasing the welding temperature from 160 °C to 200 °C, after which the load values tend to fluctuate between 17 MPa and 20 MPa with a further increase in the temperature. Therefore, the compatibilizer efficiency increases rapidly with increasing temperature. At 160 °C, which is below the melting point of PA12, the chain mobility was restricted, which, in turn, cannot induce the interaction of chain molecules [25]. At this temperature (160 °C), the joint of the interposed PA12 sheet and the EMG composite was broken at the little strength of 0.06 MPa due to insufficient reaction [26]. However, at 200 °C, as PA12 melting occurs [27], the welding strength of PMMA-PA12 by GMA reached 18 MPa, which is 300 times higher than the welding strength of PMMA-PA12 by GMA at 160 °C. This shows that fusion bounding between PMMA and PA12 by interfacial compatibilization was successfully achieved under hot-plate welding at 200 °C.

### 3.2. Fractography

**Figure 8:**
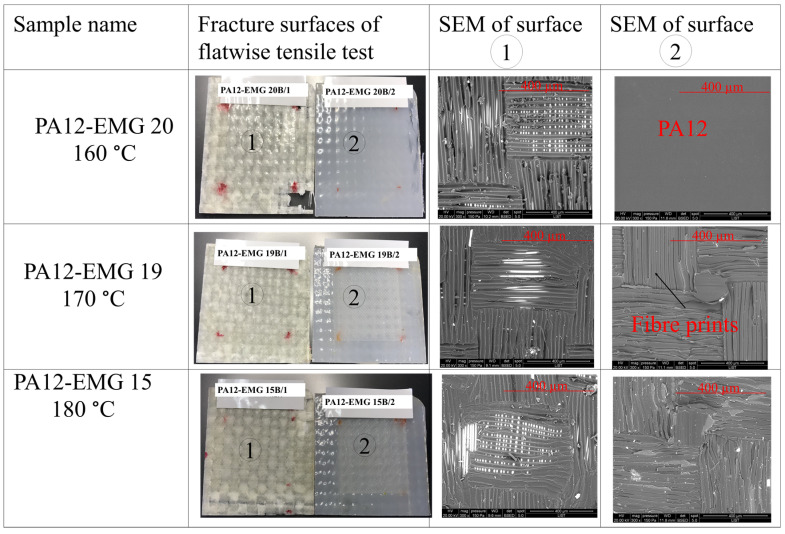
Fracture surfaces and SEM images of PA12-EMG specimens welded at temperatures of 160 °C, 170 °C, and 180 °C. The surface 1 stands for the EMG composite and the surface 2 stands for the interposed PA12 sheet.

**Figure 9:**
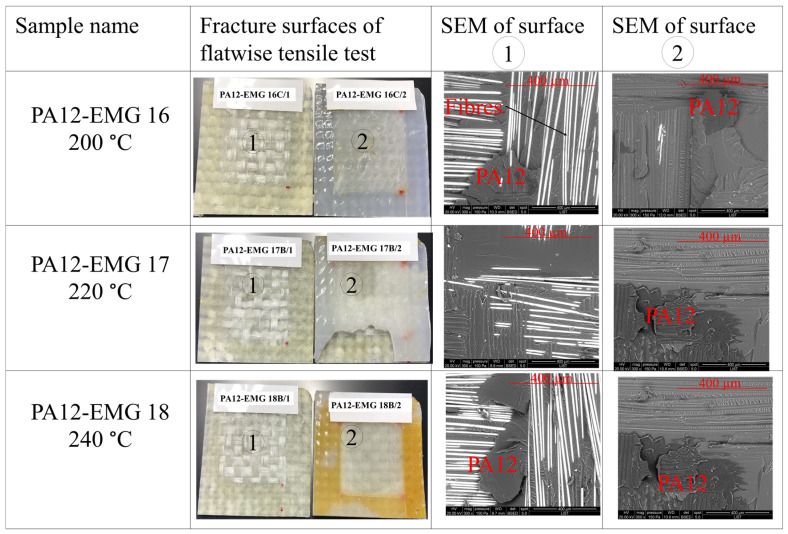
Fracture surfaces and SEM images of PA12-EMG specimens welded at temperatures of 200 °C, 220 °C, and 240 °C. The surface 1 stands for the EMG composite and the surface 2 stand for the interposed PA12 sheet.

The SEM images of the flatwise tensile fractured surfaces of specimens are shown in Figure 8 and Figure 9. The fractographic analyses revealed remarkable differences on the fracture profiles between the specimens welded in the temperature range of 160 °C to 180 °C (Figure 8) and those of 200 °C to 240 °C (Figure 9).

For the case of hot-tool reactive welding at 160 °C, Figure 8-first row shows that the surface of the adhesive is smooth and debonding occurred between the interposed PA12 sheet (labeled with number 2 in Figure 8) and the adherent (composite which is labeled with number 1). Therefore, the major fracture type is adhesive or interfacial [28]. This poor bonding quality is caused by the insufficient welding temperature. The temperature is below the melting point of PA12, and thus cannot induce the mobility of chain molecules to react with the compatibilizing agent [29].

On the other hand, the surface of the specimens welded at 170 °C in Figure 8-second row was rather rough as compared to the case of 160 °C. This is obviously due to the chain mobility of PA12. As the temperature increased to 180 °C (Figure 8-third row), the surface become rougher. The higher temperature indicates that there is the interaction of molecules at interface thanks to further mobility in the PA12 chain. It should be noted that the failure mode for the reactive welding in the temperature range of 160 °C to 180 °C was an adhesive fracture. This is well correlated to an insufficient temperature for the perfect bounding of the joints.

The perfect bounding joints are observed in Figure 9, for the welding cases at 200 °C, 220 °C, and 240 °C. The major fracture type for the cases welds at 200 °C (Figure 9-first row) or above (Figure 9-second and third rows) is a cohesive fracture. The fractured layer (PA12, marked on the SEM images) remained on the surface of both adherend (Elium^®^ composite plates) and the interposed PA12 sheet after debonding. Compared to the previous cases, the temperature for the cases in Figure 9 is higher, indicating the strong interaction at interfaces. Once the temperature increases to the melting temperature, the polymer flows under the compression force across the interface. Hence, the flowing polymer can react with GMA, and thus the composites and the interposed PA12 sheet can be bounded completely at 200 °C, thereby resulting in a weld [3]. However, there is no remarkable change on the surface of the fractured specimens with increasing temperature from 200 °C to 240 °C. Therefore, the desired type of fracture, which is cohesive [13], was achieved at a welding temperature of 200 °C and further increase will not change the bounding quality. This observation is supported with the results of flatwise tensile tests which were reported in the previous section.

## 4. Conclusions

The weldability of Elium^®^ composite copolymerized with a glycidyl methacrylate (GMA) and PA12 as an interposed sheet were investigated by hot-plate welding. PMMA and PA12 are incompatible for welding. The results of our study on the possibility of the fusion bounding for incompatible polymers by hot-plate welding showed the following:

(1) Despite the lack of knowledge on the selection of appropriate compatibilizing agent, this study proved the effectiveness of GMA on the welding of PMMA/PA12.
(2) The highest weld strengths between the incompatible polymers PA12 and Elium^®^ (PMMA) composite are achieved at a temperature above the melting temperature of PA12 and especially at 200 °C.
(3) The PMMA-GMA-PA12 copolymer has a key role in the enhancement of the interfacial adhesion between PMMA and PA12.

In future studies, the fusion bounding model for PMMA-PA12 interfaces will be proposed. In addition, promoting the formation of a better welding interface after consolidation will also be studied. Analysis of variance (ANOVA) is also helpful to analyze the influence of the processing pressure and hold time on the mechanical performance of resulting welded joints.

## Figures and Tables

**Table 1:** Temperature changes during the hot-plate welding of reactive PA12-EMG at constant hold time and constant compaction pressure. The acronym of EMG stands for Elium^®^-GMA-Glass composite. The samples are compressed with a compaction pressure of 0.5 MPa and held for a period of 10 min.

Sample Name	Heating Temperature (°C)
PA12-EMG 20	160
PA12-EMG 19	170
PA12-EMG 15	180
PA12-EMG 16	200
PA12-EMG 17	220
PA12-EMG 18	240

## Data Availability

Not applicable.
